# Isolating motile sperm cell sorting using biocompatible electrospun membranes

**DOI:** 10.1038/s41598-022-10042-0

**Published:** 2022-04-11

**Authors:** Roberto D. Katigbak, Ludovic F. Dumée, Lingxue Kong

**Affiliations:** 1 Institute for Frontier Materials, Deakin University, Waurn Ponds, Geelong, VIC 3216 Australia; 2 Department of Chemical Engineering, Khalifa University, Sas Al Nakhl (SAN) Campus, Abu Dhabi, United Arab Emirates

**Keywords:** Biological techniques, Materials science

## Abstract

Motility is an indicator of sperm cell viability due to higher probability in swimming through the female reproductive tract and undergo fertilization with the egg cell. Centrifugation method is a technique to process high volume semen and isolate motile sperm cells but decreases the biochemical integrity of spermatozoa due to the contact with reactive oxygen species (ROS) from dead cells released during centrifugation. This study uses solution electrospun poly(ε-caprolactone) membranes as an alternative in isolating motile spermatozoa by utilizing a rationally designed 3D printed module set up, providing the same benefits as commercially available techniques with minimal processing time, and bypassing the centrifugation step to provide higher quality sperm cells. The membranes, with nominal pore size distributions ranging from 5 to 6 µm are highly porous structures suitable for establishing baseline data for sperm cell sorting by motility. The proposed method allows for isolation of motile sperm cells with 74% purity, while decreasing the processing time by 98% when compared to centrifugation techniques. This novel approach provides a facile method for isolating motile spermatozoa directly from frozen semen samples without any pretreatments and is easily scalable for small and medium scale farms as well as larger industries.

## Introduction

Sperm cell quality in relation to high success rate in fertilization can be attributed to a variety of physical and biochemical characteristics^1^. Physical characteristics of high quality spermatozoa would include sperm motility, which may be described as high velocity parameters and flagellar beats^2^ and from the absence of morphological abnormalities within sperm cell head and/or tail^3^. Desired biochemical properties of sperm cells for fertilization include sperm cell maturity, evaluated from zeta potential^4^ and hyaluronic acid binding proteins present on the cell membrane^5^, and from the absence of apoptotic factors^6^. Methods to isolate sperm cells based on physical and biological properties have been developed relying on the characteristics of spermatozoa required for assisted reproductive technologies (ARTs)^7^.

Motility is one of the primary characteristics in measuring sperm cell viability and success rate in traversing through the female reproductive tract^8^ or other ARTs. There have been increasing demands in isolating sperm cells based on motility, finding its niche in the healthcare field to bypass male infertility^9^ or maintaining sire lines for high value animals and endangered species^10^. The current methods in isolating motile sperm cells from semen samples include centrifugation techniques such as swim up method and density centrifugation techniques^11^. Swim up method is where the sperm cells are centrifuged down and redispersed in solution and where fast moving sperm cells are able to “swim up” from the pellet and be isolated. In density centrifugation techniques, the semen samples are placed on top of the solutions with different densities and the highly motile spermatozoa move across more viscous liquids and subsequently are isolated^12^. Both techniques are high throughput, allowing for a quick isolation and can be done with a large number of sperm cells. However, both methods may also damage the biochemical and DNA integrity of the spermatozoa by exposure to reactive oxygen species (ROS) released by a subpopulation of cells during the centrifugation step^13^. Another route gaining popularity in sorting sperm cells by motility relies on the use of microfluidic devices, whereby fast moving spermatozoa are isolated by taking advantage of the ability of motile sperm cells to swim against the flow of the solvent, allowing for separation of live sperm cells from immature or dead sperm cells as well as other types of cells. Microfluidic methods are able to yield fast moving spermatozoa at high purity, but suffer from sample size processing issues related to the complexity in scaling up such devices^14^.

Membranes are currently being explored in different sperm cell sorting process for their wide applicability in different separation methods as their pore size can be tailor designed using various membrane synthesis processes such as electrospinning^15^ or other available commercialized techniques^16^. The use of polycarbonate membranes in electrophoresis was demonstrated whereby the sperm cells are separated based on their surface charge and the filter is used to prevent backflow of spermatozoa or other cellular components in the solution^17^. Nanofiber membranes were also introduced in a complex device to sieve off sperm cells from extracted DNA, aiding in identification of suspects in sexual assault cases as funded by the US Department of Justice^18^. Commercially available fibrous poly(ester) membrane devices (Pall Biosupport Corporation) were attached to a pipette tip, allowing for the isolation of motile sperm cells from semen samples and the separation of other types of cells and morphologically abnormal sperm cells^16^. Poly(carbonate) track etched (PTCE) membranes with cylindrical pores have also been successfully used to isolate motile spermatozoa from semen sample^19^. These reports in isolating motile spermatozoa propose different techniques with different configurations but do not address the role of membranes in the separation process, particularly exploring different filter characteristic for optimal isolation. Understanding the role of filters in sperm cell sorting allows for proper optimization not just in membrane morphology and characteristics, but also process parameters.

The goal of this study is to present a novel and standard method in isolating motile spermatozoa using electrospun poly(ε-caprolactone) (PCL) membranes, starting from the rationale design of modules for separation to establishing protocols in sperm cell isolation and assays for determining the effect of filter properties to separation efficiency and processing time. The analysis relied on the evaluation of strategic parameters such as the purity of the isolated cells and the processing time of the sorting technique.

## Results

### Poly(ε-caprolactone) electrospun membranes properties

**Figure 1:**
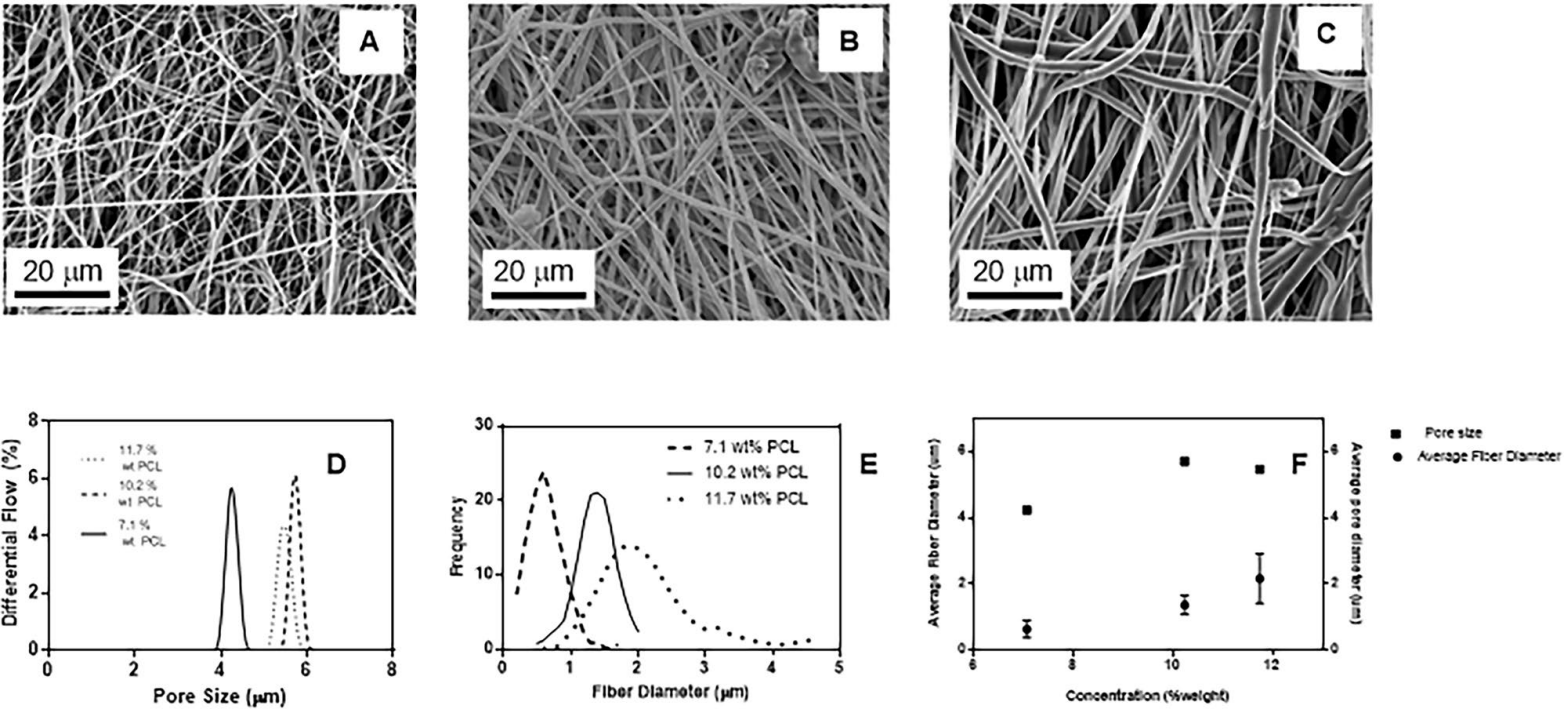
(Top) SEM images of PCL membranes obtained from electrospinning (**A**) 7.1, (**B**) 10.2 and (**C**) 11.7 wt% solutions. (**D**) Pore size distribution for the PCL membranes as determined by capillary flow porometer. (**E**) Distribution of fiber diameters for 7.1 (---), 10.2 (—) and 11.7 (···) wt% PCL membranes. (**F**) Relationship of fiber diameter and pore size to concentration of polymer solution in electrospinning of PCL.

Electrospinning may be used to fabricate highly porous membranes with pore size distributions ranging from sub–micron to sub–millimeter levels^20^. PCL was chosen as the base polymer since highly biocompatible and stable for a variety medical and cellular applications^21^. In the context of bovine sperm cell sorting, a pore size range of 5–6 µm^22^ was required to allow the spermatozoa head to diffuse and be separated from epithelial cells and leukocytes, which sizes lay around ~ 15 and ~ 30 µm, respectively^23^. The pore size of electrospun membranes may be varied with various process parameters adjustments but for initial screening in this study, the concentration of PCL polymer in solution was varied while maintaining all other parameters constant. The impact of different polymer concentrations on the fiber diameters and pore size distribution is shown in Fig. 1, where increasing the polymer concentration increases both fiber diameter and pore size as seen in other instances^24^. The pore size of the polymer was determined using capillary flow porometer where the plot of differential flow, which is the percentage of the liquid that passed through the pores, was plotted against pore size. A higher differential flow indicates a greater number of pores against the corresponding diameter. It was also observed that the mean pore size does not increase anymore going from 10.2 to 11.7 wt%, which suggest that other parameters must be adjusted in order to obtain pore sizes higher than 6 µm. For bovine sperm cell sorting, membranes with pore sizes must be greater than that of bovine sperm cell width, which is approximately 5 µm^22^. Therefore, PCL membranes spun at 10.2 wt% concentration with mean pore size of 5.7 µm were used for the succeeding experiments.

### Diffusion times

**Figure 2:**
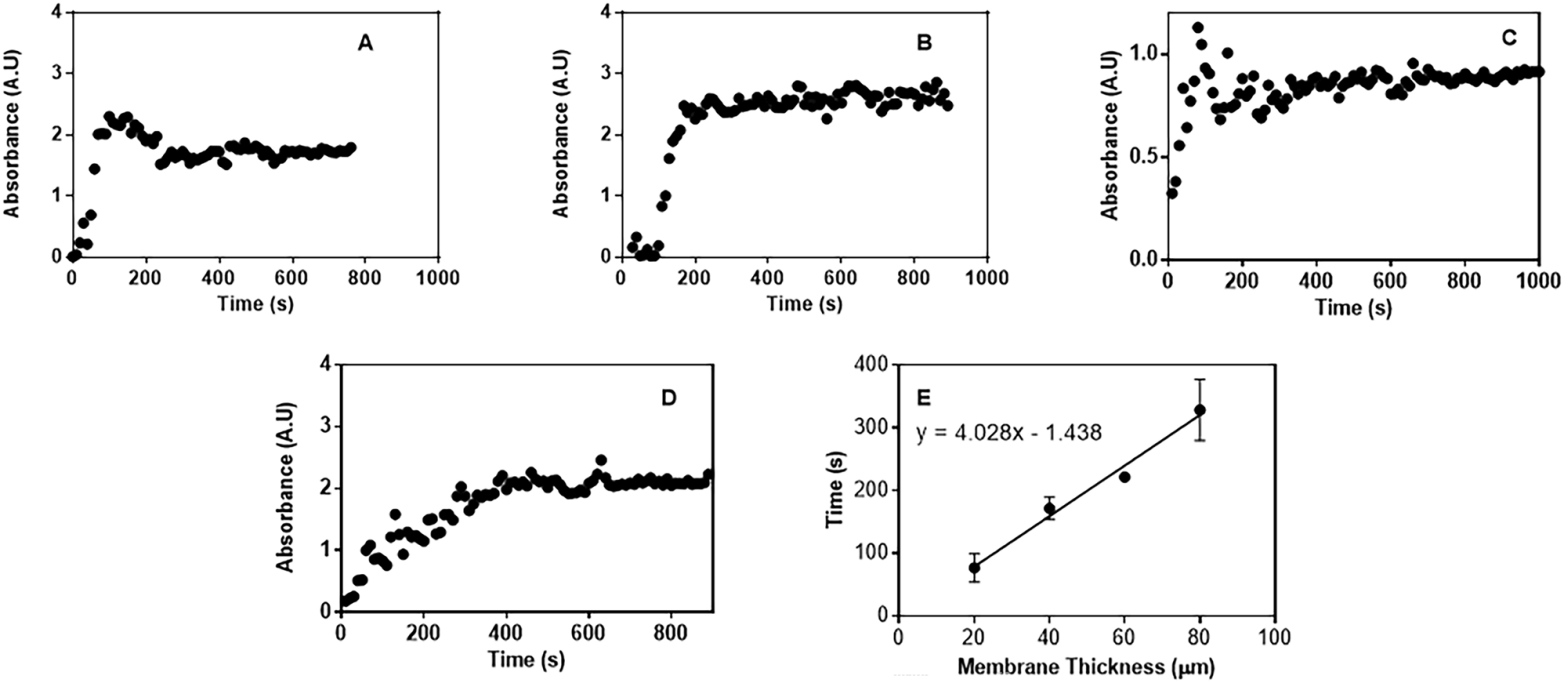
Time course diffusion of bull sperm cells using solution electrospun PCL membranes with different thickness (**A**) 20 µm (**B**) 40 µm and (**C**) 60 µm and (**D**) 80 µm. (**E**) is the graph that illustrates the relationship between calculated time required to achieve maximum diffusion (processing time) in dependence to membrane thickness (with a high *R*^2^ of 0.983 from the linear regression of experimental data).

The time taken to isolate motile sperm cells from raw semen sample is an important factor to consider whether the proposed method is comparable to commercially available techniques in semen processing. Short processing times for semen samples lessens the probability of sperm cell damage through a variety of pathways or absence of stimuli that might inhibit degradation^25^. The time to achieve maximum diffusion was determined by using the continuous assay and by monitoring the number of cells that diffused across the membrane at 300 nm. Figure 2 summarizes the effect of varying thickness of solution electrospun PCL membranes on the time required to achieve maximum diffusion. To determine the concentration of sperm cells, maximum absorbance can be observed between 280 and 300 nm. Therefore, any wavelength from this region can be used.

### Separation efficiency

The efficiency of the proposed membrane process in isolating motile spermatozoa from semen sample was evaluated using the discontinuous assay. This assay allows for simultaneous processing of thawed semen sample with multiple replicates using solution electrospun PCL membranes. The data of motile sperm cell isolation using solution electrospun PCL with 20 µm thickness are summarized in Table 1.

**Table 1:** Summary of data obtained from initial experiments with *N* = 6.

	Average values ± SEM
Membrane thickness (µm)	20 ± 0
Initial number of cells (in Millions)	10.56 ± 0.34*
Number of cells diffused (in Millions)	2.50 ± 0.51*
Percent of cells diffused (%)	23.71 ± 4.89
Average percent of motile sperm cells in Initial sample (%)	31.71 ± 2.21*
Number of motile sperm cells in initial sample (in Millions)	3.35 ± 0.108
Average percent of motile sperm cells after diffusion (%)	74.20 ± 3.29*
Number of motile sperm cells after diffusion (in Millions)	1.86 ± 0.39

The SEM for the other values were calculated by propagating the errors obtained from data with *.

**Figure 3:**
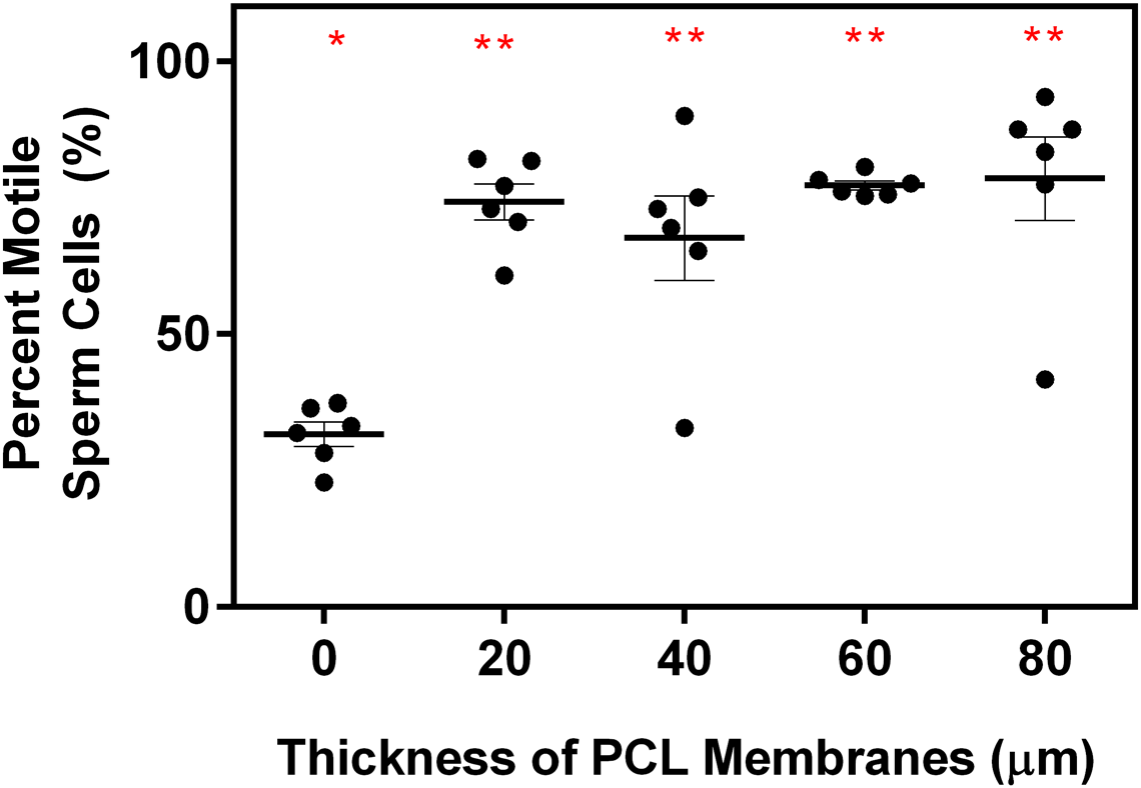
Motile sperm cell population isolated from unfiltered and solution electrospun PCL membranes with different thickness (*N* = 6). Asterisk above show groups with no significant differences in means as evaluated using one way ANOVA and Tukey’s multiple comparison test.

The interactions between the base polymer and the sperm cells were examined by evaluating the population of motile sperm cells post filtration using solution electrospun membranes with different thicknesses. These interactions would give an insight on the biocompatibility of the membrane to sperm cells. As shown in Fig. 3, it is interesting to find that within the thickness range (20–80 µm) studied, the performance of the membranes was quite stable. The appropriate thickness shall be decided by considering other factors such as pressure drops during separation with thicker membranes which will be further studied. No change in sperm cell quality post separation using thicker membranes signifies that even though solution electrospun PCL membrane does not allow non-motile spermatozoa to pass through, it does not impede motile sperm.

## Discussion

**Figure 4:**
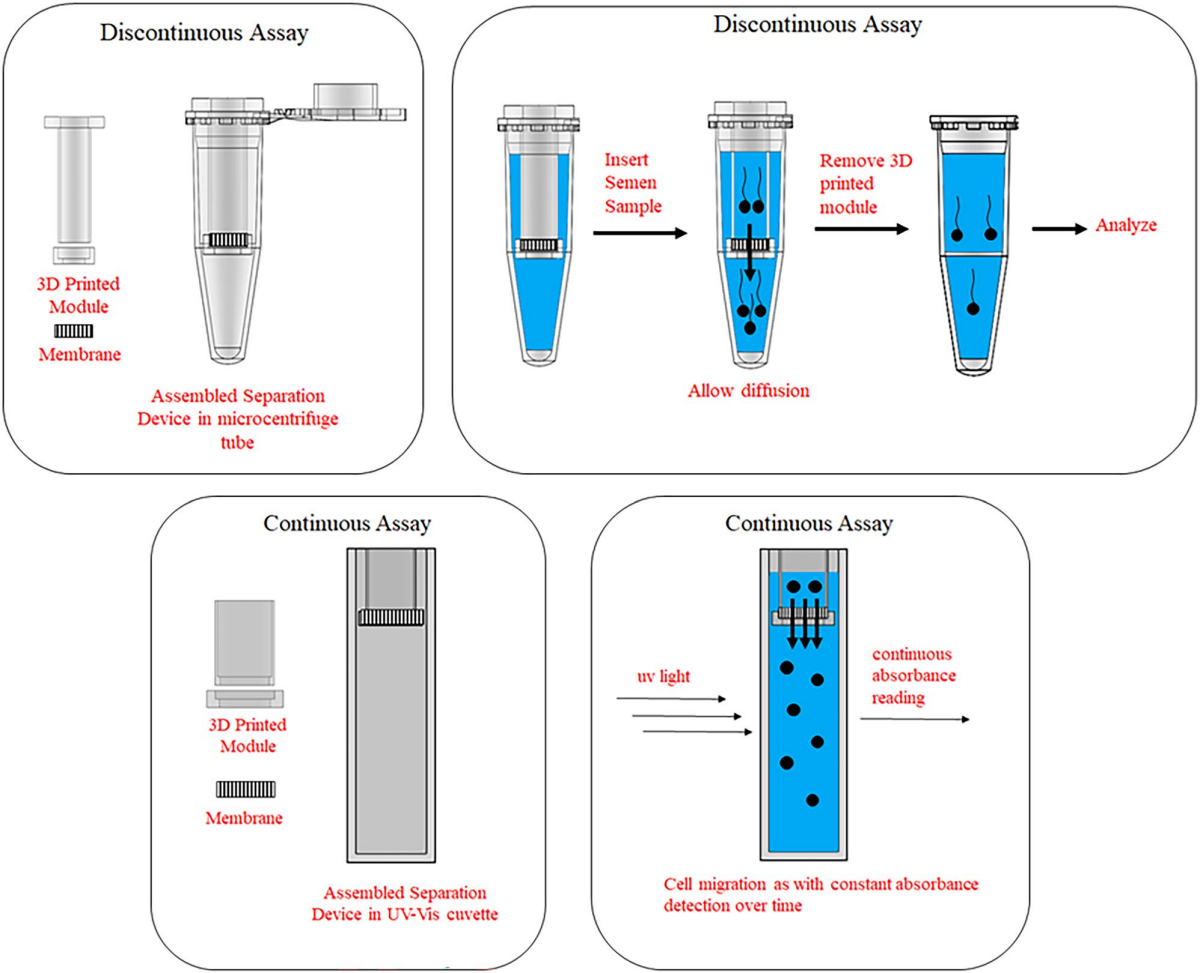
Designed 3D printed modules and their intended vessels. The 3D printed module for the discontinuous assay is utilized in a microcentrifuge tube, while cuvette is used for the continuous assay. Process diagrams are show per respective assays.

The design of separation modules requires certain parameters to be considered towards efficient separation and technology translation to an industrial scale. This includes (1) ease in scalability for high throughput separation (2) the use of biocompatible material to reduce damage to sperm cells during the isolation (3) module that can be manipulated easily by users with or without proper technical background. These requirements can be met by combining 3D printed modules using a known biocompatible polymer, poly(lactic acid)^26–28^, with common plastic wares such as centrifuge tubes and cuvettes. The chambers were initially designed for small scale separation, and the different configurations were applied to varying experiments depending on the data required (Fig. 4). In contrast to previous reports where diffusion occurs horizontally^19^, the current design takes advantage of the gravity as the diffusion occurs vertically. This design was conceptualized so that diffusion would occur faster and prevent backflow of sperm cells which had already crossed the membrane. The prevention of backflow decreases the chances of mixing the sperm cells that already diffused in the permeate and feed side due to osmotic pressure which can influence the direction at which the sperm cells swim, thereby increasing the purity of motile sperm cells collected. The module for discontinuous assay is used to compare different membranes and membrane properties at a single time point, with the chamber easily removable from the micro-centrifuge tube to access the sperm cells that traversed across the filter. Meanwhile the 3D printed accessories for the continuous assay allow for constant detection of sperm cells that are able to diffuse across the membrane using a cuvette and a UV–Vis spectrophotometer, allowing for time resolved data experiments. The use of both set ups allows for the determination of both the isolation purity of motile spermatozoa and the time required to achieve maximum diffusion of sperm cells, which are indicators of overall efficiency of the separation process. The materials in the proposed separation process are also widely available and easily scalable to larger volumes of samples, which is ideal when the technology is translated to an industrial scale.

As can be seen from the time course plots (Fig. 2), the diffusion behavior of sperm cells across the solution electrospun PCL membrane is constant, indicative of constant diffusion then plateaus at a certain absorbance. This signifies that diffusion of sperm cells is unidirectional if no pressure is applied. This mechanism gives insight that diffusion is not concentration limited where equilibrium is achieved through constant movement of cells across the membrane, but of a different parameter. This characteristic inherent to sperm cells is proposed to be high motility with only a fraction of the total population of spermatozoa can actively traverse across the membrane. Another observation is that the time required to cross the membrane increases linearly with respect to thickness, implying that stacking of the same solution electrospun PCL filters does not alter significantly the path length at which the sperm cells traverse. The observed linear increase of processing time with respect to thickness, while keeping all other factors constant, also indicates that fouling of cells or any other components of the thawed semen sample does not impede spermatozoa diffusion and sperm cell diffusion is only dependent on the increase in mean free path of the sperm traversed within the boundaries of the separation conditions. Comparing this method to commercial centrifugation techniques^29^, there as a significant decrease in processing time in the proposed method, which can achieve maximum diffusion at 77 s using 20 µm solution electrospun PCL membrane with the designed configuration while Percoll method requires 3600 s. The proposed method also eliminates the need for a centrifugation step, which preserves the integrity of the sperm cell by avoiding the exposure to reactive oxygen species.

Another conclusion that can be obtained from the proposed method is that the number of sperm cells diffusing across the membrane coincides with the number of motile spermatozoa in the original semen sample (Table 1). The values of motile spermatozoa in original semen and number of sperm cells isolated further validates that it is the spermatozoa motility that allows the cells to traverse the solution electrospun PCL membrane, allowing for the isolation of fast swimming sperm cells from thawed semen.

One important note from the experiments done is that the number of motile sperm cells post processing is lower than the number of cells that diffused, which indicates that the overall process has a degree of degrading effect on sperm cells based on motility. The mechanism is still unclear as to how the solution electrospun PCL membrane inhibit the diffusion of non-motile sperm cells, but it can be postulated that the filter’s inherent tortuous morphology can trap the non-moving cells, allowing for the isolation of fast swimming spermatozoa. Even though the membrane traps non-motile sperm cells, there are no evidences that this affects isolation efficiency which can be attributed to the highly porous nature of solution electrospun PCL membrane. Nevertheless, the proposed method in isolating viable sperm cells is comparable to that of already commercially available techniques^29,30^ where the post processed samples contain approximately around 70% motile semen sample, improving overall the number of viable cells which can be used to increase the success rate of different ARTs. It can be then concluded that thicker solution electrospun PCL (up to 80 µm, Fig. 3) does not require spermatozoa to expend more energy ^31^ when traversing, therefore does not decrease overall number percentage of motile sperm cells. No change in percent motility in increasing membrane thickness is also indicative that the biocompatibility of the PCL with sperm cells is not the cause for the presence of non-motile sperm cells in the solution post separation on the premise that only motile spermatozoa can pass through the membrane. These results also indicate that when the thickness increases from 20 to 80 µm, there is no significant change in the flow within the membranes. However, further studies should be done by measuring the pressure drop during separation and undertaking theoretical analysis such as computational fluid dynamics (CFD) to determine effects of fouling on the membrane. Since the permeate side contains approximately 25% non-motile sperm cells, the loss of motility of previously motile spermatozoa can be delineated to other aspects in separation process such as temperature control or sudden change in swimming environment. Overall, thinner solution electrospun PCL membrane is still desired for sperm cell sorting by motility, since it has lower processing time than thicker filters without significant increase in purity of motile sperm cells. Thin membranes will increase overall efficiency and decrease the chances of exposing sperm cells to external factors which can affect biochemical integrity.

## Methodology

### Materials

Poly(ε-caprolactone) (MW = 80 kDa), AR grade chloroform, HPLC grade methanol, sodium hydroxide, pre mixed phosphate buffer saline at pH = 7.4 and bovine serum albumin were purchased from Sigma Aldrich (Australia). Bull semen samples are obtained for Total Livestock Genetics (Australia). Modules were 3D printed using Original Prusa i3 MK2s printer with filament poly(lactic acid) (PLA) from Prusa Research (Czech Republic).

### Fabrication and characterization of PCL membranes

Electrospinning of PCL membranes was achieved in a HOLMARC HO-NFES-043U (Kalamassry, Kerala, India). Different concentrations of PCL membranes (7.1, 10.2 and 11.7 wt%) for solution electrospinning were made by dissolving the appropriate weight of PCL pellets in 25% methanol in chloroform by sonication. The PCL solutions were spun using 5.0 mL syringe and 18 gauge needle, at 25 °C, 11 kV, 1 mL/h flow rate and 12 cm distance from needle tip to mandrel collector at 800 rpm speed for 90 min.

Scanning electron microscope images were obtained using Jeol NeoScope JCM 5000 (Japan) at 5 kV. Prior to SEM imaging, membranes are coated with 5 nm gold particles using a BAL–TEC SCD 050 sputter coater (Leica Microsystems, Australia). Pore diameters of 20 µm thick membranes were estimated using capillary flow porometer (3G Zh Quantachrome, Florida, USA) with pressure between 0.04186 to 0.1674 bar. Fiber diameters were calculated using imageJ.

### Determination of sperm concentration

Two frozen semen samples were thawed at 37 °C for 1 min and diluted in 3% (w/v) NaCl aqueous solution as seen in Table 2. The absorbance at 280 nm of all the replicate solutions were measured three times using a UV–Visible spectrophotometer (NanoDrop 2000C, ThermoFisher Scientific, USA) and were averaged for each replicate. The total number of replicates per sample is *N* = 6.

**Table 2:** Sample dilution of semen sample for calibration curve.

Sample number	Semen volume (µL)	Volume of 3% (w/v) NaCl (µL)	Total volume (µL)
1	5	45	50
2	4	46	50
3	3	47	50
4	2	48	50
5	1	49	50

In order to determine sperm cells concentrations, 10 µL aliquots were diluted 10-folds (sample number 1) and mounted in a haemocytomer with 0.10 mm height, to allow for cell counting under a Zeiss Axio Lab.A1 phase contrast microscope. Each cell counting was done twice for each replicate, with the total number of *N* = 6. The cell count from sample number 1 was used to calculate the initial concentration of semen sample, which was used to obtain the sperm concentration in terms of million cells per mL in the succeeding diluted solution. Linear regression was used to determine the relationship of absorbance measurements and sperm cell concentration, where the *y*-intercept is forced to 0 to account that there are no sperm cells at 0 absorbance.

### Determining time for maximum diffusion

PCL membranes were fabricated using 10.2 wt% dope concentration for the succeeding experiments. The initial membrane had a thickness of 20 µm as measured with a micrometer caliper and different final membrane materials thicknesses were achieved by stacking several 20 µm together 2–4 times, to achieve 40, 60 and 80 µm in overall thickness. Stacking of thin membranes was chosen so that the inner structure won’t significantly be changed in contrast to fabricating thick membranes.

The membranes were placed in a 3D printed module and mounted in cuvettes filled with 2.5 mL 0.3% (w/v) BSA in PBS buffer, pH = 7.4 and were equilibriated at 37 °C prior to performing the assay. The Nanodrop UV–Visible spectrophotometer at 37 °C with wavelength detection set at 300 nm was used to detect the diffused sperm cells for 900 s or longer. Time required to achieve maximum diffusion was calculated by taking the best fit line corresponding to the plateau and the steady increase in absorbance and calculating the point where *x* intercepts both equations.

### Standard procedure for isolating motile sperm

Six frozen semen samples from six different bulls were thawed at 37 °C for 1 min and pooled prior to sperm cell sorting. In this experiment, the total number of replicate membranes is *N* = 6. Solution electrospun PCL membranes were mounted on the 3D printed module, which was allowed to achieve equilibrium at 37 °C in 1 mL 0.3% (w/v) bovine serum albumin (BSA) in phosphate buffer saline (PBS) at pH = 7.

After achieving equilibrium, 100 µL aliquots of semen samples were pipetted inside the module and diffusion was allowed to occur for 30 min. The module was then removed and the semen sample was analyzed by measuring the absorbance at 280 nm and the number of motile sperm cells was counted by evaluation under a phase contrast microscope. For a blank sample, 100 µL of semen is diluted with 1 mL of 0.3% (w/v) BSA in PBS at pH = 7.4. Further dilution was done by diluting 100 µL of blank sample in 100 µL of 0.3% (w/v) BSA in PBS at pH = 7.4, which was the solution used to calculate initial concentration of sperm cells in frozen semen sample by measuring the absorbance and evaluate the number of motile sperm cells in population under a phase contrast microscope. Absorbance measurements were done in triplicate and counting of motile sperm cells was done in duplicate measurements, each measurement used the total sperm cells in five different field of view under the microscope.

### Statistical analysis

Significant differences in mean for isolation purity were determined through one way analysis of variance (ANOVA) and Tukey’s multiple comparison analysis. Normality was determined using the Kolmogorov–Smirnov Test with Dallal–Wilkinson–Lillie for *P* value and Browne–Forsythe test for homogeneity of variance.

## Conclusions and prospects

This study proposes a new method in isolating motile sperm cells from semen sample without the use of centrifugation, which affects the integrity of spermatozoa by exposure to radical oxygen species excreted by dead cells. The technique utilizes the ability of motile sperm cells to diffuse through a solution electrospun PCL membrane and allows for identifying key parameters in evaluating the efficiency of the separation parameters such as purity and processing time. It was seen that the purity of isolated motile sperm cells is comparable to commercially available centrifugation techniques, with the processing time decreasing drastically by 98% by using 20 µm solution electrospun PCL membranes. This study establishes a new protocol which can be used in the laboratory setting to further identify efficiency parameters when optimizing membranes for sperm cell sorting. The rationally designed membrane process is also easily translatable to an industrial scale, requiring low capital and expertise to maneuver especially for small to medium scale farmers or health centers.
